# Supplementary material for: Investigation on Electrospun and Solvent-Casted PCL-PLGA Blends Scaffolds Embedded with Induced Pluripotent Stem Cells for Tissue Engineering
Source: Pharmaceutics. 2023 Dec 6;15(12):2736. doi: 10.3390/pharmaceutics15122736 (PMC10747843; doi:10.3390/pharmaceutics15122736)
Supplement: Supplementary file 1 [file pharmaceutics-15-02736-s001.zip › pharmaceutics-2688607-supplementary revised.pdf]

SUPPLEMENTARY MATERIAL

S1. Supplementary information for materials and methods and results and discussion

S1.1 Materials and methods

S1.1.1. Determination of critical entanglement concentration of PLGA and PCL solutions

To determine the critical entanglement concentration (CEC) of the used polymers, pure PCL and pure PLGA solutions at different concentrations were prepared in the DCM/DMF 75:15 solvent system. Briefly, each polymer was weighted according to the final polymer concentration (1%, 2%, 3%, 4%, 10%, 11%, 12% w/v, and only for PCL, 14% and 18% w/v) and swollen in DCM for at least 5 hours at room temperature. The solutions were vortexed every hour for 10 seconds until complete dissolution of the polymer; DMF was added, and the solutions were kept under stirring at 100 rpm for 90 minutes before being sonicated for 15 minutes to remove trapped air bubbles and proceed with viscosity measurements. A Kinexus Malvern Pro+ rotational rheometer (NETZSCH-Gerätebau GmbH, Verona, Italy) was used for rheological analyses. It was outfitted with CP1/60 and CP4/40 conical geometries, which were applied to more diluted and concentrated polymer solutions, respectively, and a solvent trap to prevent solvent evaporation during the analysis. First, the linear viscoelastic region (LVER) for each polymer solution was determined by performing an amplitude sweep test (applied shear strain between 10-1 and 103%, 1 Hz frequency, temperature 33°C), followed by a shear rate ramp analysis performed applying shear strain values within the LVER (time 5 minutes, temperature 33°C). To determine the zero-shear viscosity η0 (Pa*s), the intercept with the Y axis of the straight line in the viscosity (Pa*s) vs. shear rate (s-1) plot was calculated (Shaw, 2016). Eventually, the specific viscosity ηsp of the polymers was calculated according to Eq.S1 (Chiesa et al., 2022):

$$\eta_{sp} = \frac{\eta_0 - \eta_s}{\eta_s} \qquad \text{Eq. S1}$$

where η_s is the solvent viscosity; since the solvent viscosities were neglectable (DCM=0.437 mPa*s and DMF=0.802 mPa*s), the specific viscosity η_{sp} was equal to the zero-shear viscosity η_0. To finally assess the critical entanglement concentration, zero shear viscosity (Pa*s) and polymer solution concentration (% w/v) were plotted on a bi-logarithmic plot. Two trends were observed: a first trend with less slope describing the increase of viscosity in diluted solutions, and a second trend with greater slope describing the behaviour of concentrated polymer solutions. The intercept between the two trends is the CEC and was calculated by solving the following mathematical system:

$$\begin{cases} y_2 = c_2 * x^{b2} \\ y_1 = c_1 * x^{b1} \end{cases} \qquad \text{Eq. S2}$$

$$c_{2^*} x^{b2} = c_{1^*} x^{b1} \qquad \text{Eq. S3}$$

$$x = \left(\frac{c_1}{c_2}\right)^{\frac{1}{b2-b1}} \qquad \text{Eq. S4}$$

were y_1 is the equation describing the trend of diluted polymer solutions (below CEC) and y_2 is the equation describing the trend of concentrated polymer solutions (above CEC).

S1.1.2. Electrospinning parameters optimization

In accordance with CEC calculation, 2 polymer solutions at different concentration for each polymer type were subjected to electrospinning trials. Briefly, 12% and 14% w/v PCL, and 8% and 10% w/v PLGA solutions in DCM/DMF 75:15 solvent system were prepared as previously described in *S1 Methods, section S1.2. Electrospinning parameters optimization*. The solutions were loaded into a syringe equipped with a 25-gauge needle tip, placed at a distance of 15 cm and electrospun on a flat collector using a NANON 01A Electrospinner (MECC Co. Instruments, Fukoka, Japan). The temperature was kept between 30 and 35 °C and the relative humidity stabilized at 23%. Polymer solution flow rate and voltage were the main investigated process parameters and they were varied between 0.1 and 0.6 mL/h, and 10 to 25 kV, respectively as reported in **Table S1**. Taylor cone formation and stability, polymer jet whipping amplitude, fibre formation and adequate solvent evaporation were evaluated.

Table S1. Electrospinning trials performed on 12% or 14% w/v PCL, and 8% or 10% w/v PLGA solutions in in DCM/DMF 75:15 solvent system. Polymer solution flow rate and voltage were varied and Taylor cone (TC) formation, fibres formation, drying and electrospinning process stability are reported.

Trial n.	Polymer type and solution concentration (% w/v)	Voltage (kV)	Flow rate (mL/h)	Taylor cone (TC) formation and stability	Fibres formation and drying
1	PCL 12%	10	0.1	No TC	-
2	PCL 12%	12	0.1	No TC	-
3	PCL 12%	15	0.1	Yes TC, unstable	Frequent interruption and spraying
4	PCL 12%	10	0.2	Yes TC, unstable	Frequent interruption and spraying
5	PCL 12%	12	0.2	Yes TC, unstable	Frequent interruption and spraying
6	PCL 12%	15	0.2	Yes TC, unstable	Frequent interruption and spraying
7	PCL 12%	12	0.4	No TC, dripping	-
8	PCL 12%	18	0.4	Yes TC, unstable	Frequent interruption and spraying
9	PCL 12%	20	0.4	Yes TC	Spraying

10	PCL 12%	25	0.4	Yes TC	Spraying
11	PCL 14%	10	0.1	No TC	-
12	PCL 14%	12	0.1	No TC, dripping	-
**13**	PCL 14%	15	0.1	Yes TC, stable	Wide whipping, continuous spinning
**14**	PCL 14%	18	0.1	Yes TC, stable	Wide whipping, some interruptions
15	PCL 14%	18	0.2	Yes TC, unstable	Wide whipping, but frequent interruption
**16**	PCL 14%	15	0.3	Yes TC, stable	Wide whipping, continuous spinning
17	PCL 14%	15	0.5	Yes TC, stable	Wide whipping, continuous spinning
**18**	PCL 14%	15	0.6	No TC, dripping	-
19	PLGA 8%	10	0.1	No TC	-
20	PLGA 8%	12	0.1	No TC, dripping	-
21	PLGA 8%	15	0.1	Yes TC	Small whipping, wet fibres
22	PLGA 8%	18	0.1	Yes TC, unstable	Whide whipping, TC splitting
23	PLGA 8%	15	0.3	No TC, dripping	-
24	PLGA 10%	10	0.1	No TC	-
25	PLGA 10%	12	0.1	Yes TC	Small whipping, wet fibres
**26**	PLGA 10%	15	0.1	Yes TC, stable	Good whipping, continuous spinning
**27**	PLGA 10%	18	0.1	Yes TC, stable	Wide whipping, continuous spinning
28	PLGA 10%	20	0.1	Yes TC, unstable	Wide whipping, TC splitting
**29**	PLGA 10%	15	0.3	Yes TC	Wide whipping, continuous spinning
30	PLGA 10%	15	0.6	Yes TC, but dripping	Wide Whipping, wet fibres
**31**	PLGA 10%	18	0.3	Yes TC	Good whipping, continuous spinning
32	PLGA 10%	18	0.6	Yes TC, but dripping	Wide Whipping, wet fibres

The better performing electrospinning trials in terms of Taylor cone (TC) formation, fibres formation and drying, and overall process stability, namely trials n. 13, 14, 16, and 17 obtained form 14% w/v PCL solution,

and trials n. 26, 27, 29 and 31 obtained from 10% w/v PLGA solution, were characterized for their morphology by Scanning Electron Microscopy (SEM), as reported in *Material and Methods, section 2.2.3. Scaffolds physical-chemical characterization*.

S1.1.3. Porogen selection for solvent casting/particulate leaching optimization

Solvent casting technique was optimized through several trials, mainly focusing on the porogen composition able to achieve a scaffold with high and homogeneous porosity. All the trials were performed on 10% w/v PCL solutions in dioxane, prepared as previously reported for electrospinning solutions. Three different types of porogens were tested: sodium chloride (NaCl), glucose (Glc), and spray-dried lactose (Lac). Glc and Lac were employed as supplied without any further treatment, their particle size was between 40 and 100 μm and between 110 and 200 μm, respectively. NaCl was sieved through ASTM sieve series (Giuliani, Milan, Italy) with 710, 500, 355, 250, 180, 125, and 90 μm mesh openings, following standardised EU Pharmacopoeia procedure for granulometric evaluation of powders ("Particle size distribution estimation by analytical sieving," 2022); NaCl crystals in the range of 500 to 710 μm were selected as porogen. Briefly, the polymer-porogen mixture was prepared by adding the requested amount of porogen to the 10% w/v PCL solution, mixing thoroughly until a uniform suspension was obtained. 300 or 600 uL of the mixture were withdrawn with a Microman E Positive Displacement Pipette (Gilson Italy, Cinisello Balsamo, Italy) and uniformly distributed in a 2.5 cm diameter Teflon mould (4.9 cm2 surface), and the solvent was evaporated under a chemical hood for 16 hours. The dry polymer film was detached from the mould and the porogen removed by dialysis and lyophilized, as reported in Material and Methods, section 2.2.2. Scaffolds preparation by porous leaching /solvent casting). Dry scaffolds were morphologically analyzed by SEM, as reported in Material and Methods, section 2.2.3. Scaffolds physical-chemical characterization. Tested porogen concentrations and amounts of moulded solution are reported in Table S2.

Table S2. 10% w/v PCL solvent casting solutions compositions using different types of porogens, namely sodium chloride (NaCl), glucose (Glc) and spray dried lactose (Lac), and process parameters. Cell culture suitable morphology was assessed by SEM analyses.

Trial	Porogen type	Porogen concentration (g/mL)	Casted polymer solution volume (μL)	Solution temperature (°C)	Morphology
**A**	NaCl	1.5	300	20	Absence of pores
B	NaCl	1.5	600	20	Extremely large pores (no polymer film formed)
C	NaCl	1.5	300	40	Absence of pores
D	NaCl	1.5	600	40	Heterogeneous porosity with large pores (no polymer film formed)

**E**	Glc	0.35	300	20	Heterogeneous porosity
F	Glc	0.35	600	20	Extremely large pores
**G**	Lac	0.35	300	20	Good and uniform porosity
H	Lac	0.35	600	20	Heterogeneous porosity

S1.2. Results and Discussion

S1.2.1. PLGA and PCL critical entanglement concentration (CEC) and selection of polymer concentrations for electrospinning trials.

The lowest concentration at which polymer-polymer interactions are able to overcome a polymer solution surface tension and permit the creation of a continuous polymer jet using electrospinning is known as the critical entanglement concentration (CEC) (Haider et al., 2018). Lower concentrations result in electrospraying, whereas a polymer solution with concentration equal to CEC generally result in poor fiber formation with beading. Polymer concentration above CEC is needed to achieve smooth and continuous fibres . The critical entanglement concentration depends not only on the polymer's characteristics (molecular weight, branching), but also on solvent and environmental temperature (Ibrahim and Klingner, 2020). Hence CEC should be experimentally assess accordingly to the solution parameters. CEC can be extrapolated from rheological analyses of diluted and concentrated polymer solutions as follows: the zero-shear viscosity of each solution is plotted versus its concentration and the intercept of the two straight lines, describing viscosity trends of the diluted and concentrated polymer solutions is calculated (Chiesa et al., 2022). The results of rheological characterization of PCL and PLGA solutions are reported in **Figure S1**.

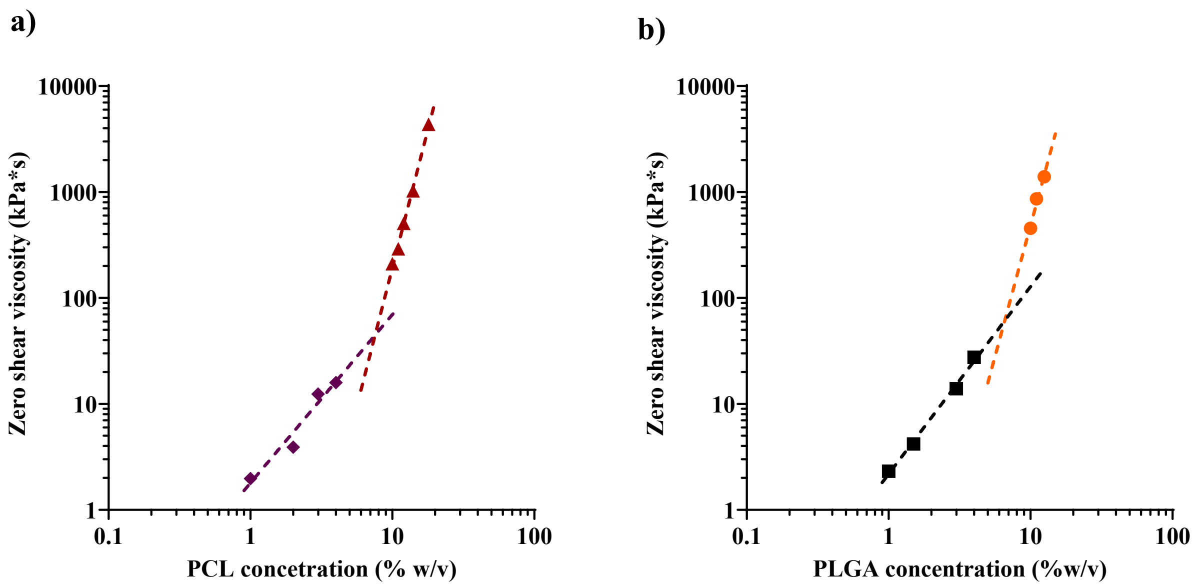

Figure S1. Zero-shear viscosity vs polymer concentration in bi-log plots of a) PCL polymer solutions and b) PLGA polymer solutions. The values are presented as average zero-shear viscosity (n=3); standard deviations are not visible on the plot because too small for the used scale. The intercepting point of the two straight lines in each point is the CEC. For PCL solution the two equations are $y_1=1.7627x^{1.5916}$ and $y_2=0.0012x^{5.2158}$, for PLGA the two equations are $y_1=2.1418x^{1.8105}$ and $y_2=0,0055x^{4,9456}$.

for the critical entanglement concentration of PCL in the selected solvent system at 33°C was 7.47% w/v, PLGA CEC was 6.70% w/v. The elevated CEC of PLGA aligns with the polymer's increased molecular weight, leading to higher viscosity values when compared to polymer solutions with the same concentration of PCL Moreover, PLGA, 14% and 18% w/v solutions couldn't be tested because the polymer was not completely dissolved, resulting in an inhomogeneous gel-like solution. In accordance with the calculated CEC, two solutions for each polymer type with concentration above CEC were selected for electrospinning trials: 12% and 14% w/v for PCL, and 8% and 10% w/v for PLGA.

S1.2.2. Electrospun trials morphological evaluation and setting of final electrospinning conditions.

The high number of parameters involved in electrospinning process, such as polymer concentration, solvent type, applied voltage, polymer solution flow rate, needle-to-collector distance, influence the final electrospun scaffold morphology. Thus, to set electrospinning variables several electrospinnig trials were performed (**Table S1**).It is well known that not only the polymer concentration has a critical value, but also other electrospinning parameters including voltage and flow rate. A critical voltage needs to be reached in order to enhance the repulsive forces inside the polymer solution droplet, to induce its deformation into a Taylor cone obtaining a polymer jet and ultrafine fibres. Moreover, the applied voltage is also responsible for increasing whipping amplitude of polymer jet, and therefor also for stretching and thinning of the emergent fibres. Nonetheless, for some polymer types it was observed that an excessive increase of voltage resulted in an increase in fibres diameter, due to a simultaneously increase also in the polymer solution flow rate. Actually, voltage has the effect to destabilize the polymer solution, due to electrical charges flow and should therefor always be matched to an appropriate flow rate, to avoid beading or breaking of the fibres. Flow rate plays also an important role in fibers morphology: dripping, wet, beaded or ribbon-like fibres are obtained if the flow rate is too high, whereas insufficient flow rate can lead to receding polymer jets with unstable Taylor cones and to the formation of fibres with wide dimensional range. Moreover, a correlation between flow rate and polymer solution surface charge density was observed: by increasing flow rate an increase of the electrical current inside the solution occurs, followed by a decrease of charge density and the merging of forming fibres (Haider et al., 2018; Ibrahim and Klingner, 2020).

The electrospinning trials were performed on two solutions at different concentrations for each polymer type. It was not possible to successfully spin 12% w/v PCL and 8% w/v PLGA solutions. 12% w/v PCL solution resulted in discontinuous and electrosprayed fibres at intermediate voltage (12 to 18 kV) and low flow rate (0.1 to 0.2 mL/h). Voltage and flow rate lowering impeded the formation of a Taylor cone, whereas an excessive

increase led to sole electrospraying. 8% w/v PLGA polymer solution could be electrospun at low flow rate (0.1 mL/h) and intermediate voltage (15 to 18 kV), but the fibres resulted either wet because of insufficient whipping and solvent evaporation, or not homogeneously distributed on the collector because of Taylor cone splitting in multiple jets and excessive whipping. Increasing or lowering the investigated parameters led to polymer solution dripping. Based on these observations, 12% w/v PCL and 8% w/v PLGA solutions were ruled out because electrospinning process was not appliable within the set up process conditions. We hypothesized the polymer concentration was insufficient to guarantee enough cohesion inside the solution to allow the formation of a Taylor cone getting a stable electrospinning process. On the other hand, 14% w/v PCL and 10% w/v PLGA solutions gave satisfactory results. 14% w/v PCL solution was electrospinnable at 15 and 18 kV and in a wide range of flow rates between 0.1 and 0.5 mL/h. Trials n. 13, 14, 16, and 17 were morphologically analyzed through SEM (**Figure S2**) and ImageJ measurements (**Table S3**). SEM images clearly showed morphological differences among the fibres of the four tested electrospinning conditions: trial n.13 displayed well-formed and regular sized fibres, even if their surface resulted slightly waved and merging of the fibres was observed in some spots of the sample; trial n.14 clearly showed broken fibres uniformly distributed on the samples surface; trial n. 16 was characterized by inhomogeneous fibre diameter, with both very thick and thin fibres, including also slighted beaded fibres; finally, trial n.17 gave the worst results, with deposition of flattened, ribbon-like, and thick fibres.

Table S3. Morphometric characterization of trials 13, 14 and 16 obtained by ImageJ analysis. Trial n.17 was excluded from morphological evaluation. Values of fibre and pore diameter are reported as average±SD. For fibre diameter measurements 45 fibre were analyzed, whereas porosity was determined using Particle analyzer tool of ImageJ software.

**Trial n°**	**13**	**14**	**16**
**Fiber diameter ∓ SD (μm)**	0.997 ∓ 0.225	0.988 ∓ 0.229	0.975 ∓ 0.553
**Pore diameter ∓ SD (μm)**	3.92 ∓ 3.41	3.15 ∓ 2.55	2.87 ∓ 2.36
**Porosity (%)**	45.54	35.57	28.09

Table S4. Morphometric characterization of trials 26, 27, 29 and 31 obtained by ImageJ analysis. Values of fibre and pore diameter are reported as average±SD. For fibre diameter measurements 45 fibre were analyzed, whereas porosity was determined using Particle analyzer tool of ImageJ software.

**Trial n°**	**26**	**27**	**29**	**31**
**Fiber diameter ∓ SD (μm)**	0.607 ± 0.107	0.668 ± 0.175	0.735 ± 0.178	1.101 ± 0.164
**Pore diameter ∓ SD (μm)**	3.62 ± 3.04	5.29 ± 6.92	5.71 ± 6.38	5.99 ± 5.34
**Porosity (%)**	51.60	71.90	71.00	66.74

In accordance with the previously mentioned effect of voltage and flow rate on fibre morphology, an voltage increase up to 18 kV at low flow rate (0.1 mL/h) resulted in excessive pulling of the PCL polymer jet with

consequent fibre rupture. On the other hand, using 15 kV of voltage but increasing flow rate from 0.3 to 0.5 mL/h resulted first in the formation of fibres with a wide range of diameters, probably due to Taylor cone destabilization leading to deposition of extremely wet fibres that flatten and merged on the collector surface in a ribbon-line fibre net. The best results were obtained applying 15 kV and 0.1 mL/h flow rate in trial n.13. It is noteworthy that the PCL fibres still have a wavy surface, probably due to insufficient stretching and drying; this could be addressed by increasing the needle-to-collector distance and so the travelling time of polymer jet, but this arrangement was not possible because of instrumental limitation (max. needle-to-collector distance 15 cm). Regarding fibre diameter (Table S3), trial n. 13, 14, and 16 did not show significant differences and were all around 1 μm; trial n.17 was excluded from morphometric evaluation because of absence of well-formed fibres. Trial n.16 showed significant lower pore diameter and overall porosity, possibly due to the highly irregular shaped and unevenly distributed fibres. 10% w/v PLGA polymer solution was also electrospinnable between 15 and 18 kV and at a flow rate between 0.1 and 0.3 mL/h. Trials n. 26, 27, 29, and 31 were morphologically analyzed through SEM (**Figure S3**) and ImageJ measurements (**Table S4**) were performed. This type of solution performed overall better than 14% w/v PCL solution, and in all the analyzed trials, fibres were well formed, had a smooth surface and were very thin (below 1 μm).

Trial n.31 significantly differs ($p<0.0001$) in fibre diameter from the other three electrospun trials, whereas trials n. 26, 27 and 29 had all comparable fibre size, meaning that the combination of higher voltage (18 kV) and higher flow rate (0.3 mL/h) led to an increase of fibre diameter, due to the solution pulling effect of the higher voltage. Regarding porosity, trial n.26 had the smaller average pore size of all electrospun PLGA matrixes ($p<0.0001$), having therefor the higher amount of micropores. On the other hand, trials n. 27, 29 and 31 reported larger pores but a high standard deviation, due to the presence of both a high number of large pore and high amount of micropores. The highest porosity percentage values were recorded for trials n. 27 and 29. The lowest porosity percentage and small average pore size of trial n.26 might indicate less spreading of the fibres on the collector, leading to a higher fibre density and hence to a reduction of empty space.

According to morphological characterization of electrospinning trials, the electrospinning parameters of trial n.16 and trial n.26 were used, namely a voltage of 15 kV and a feed rate of 0.1 mL/h. These parameters were selected in order to obtain continuous, smooth fibres with average diameter below 1 μm; moreover, by applying this settings, polymeric mats with comparable average pore size and overall porosity can be obtained.

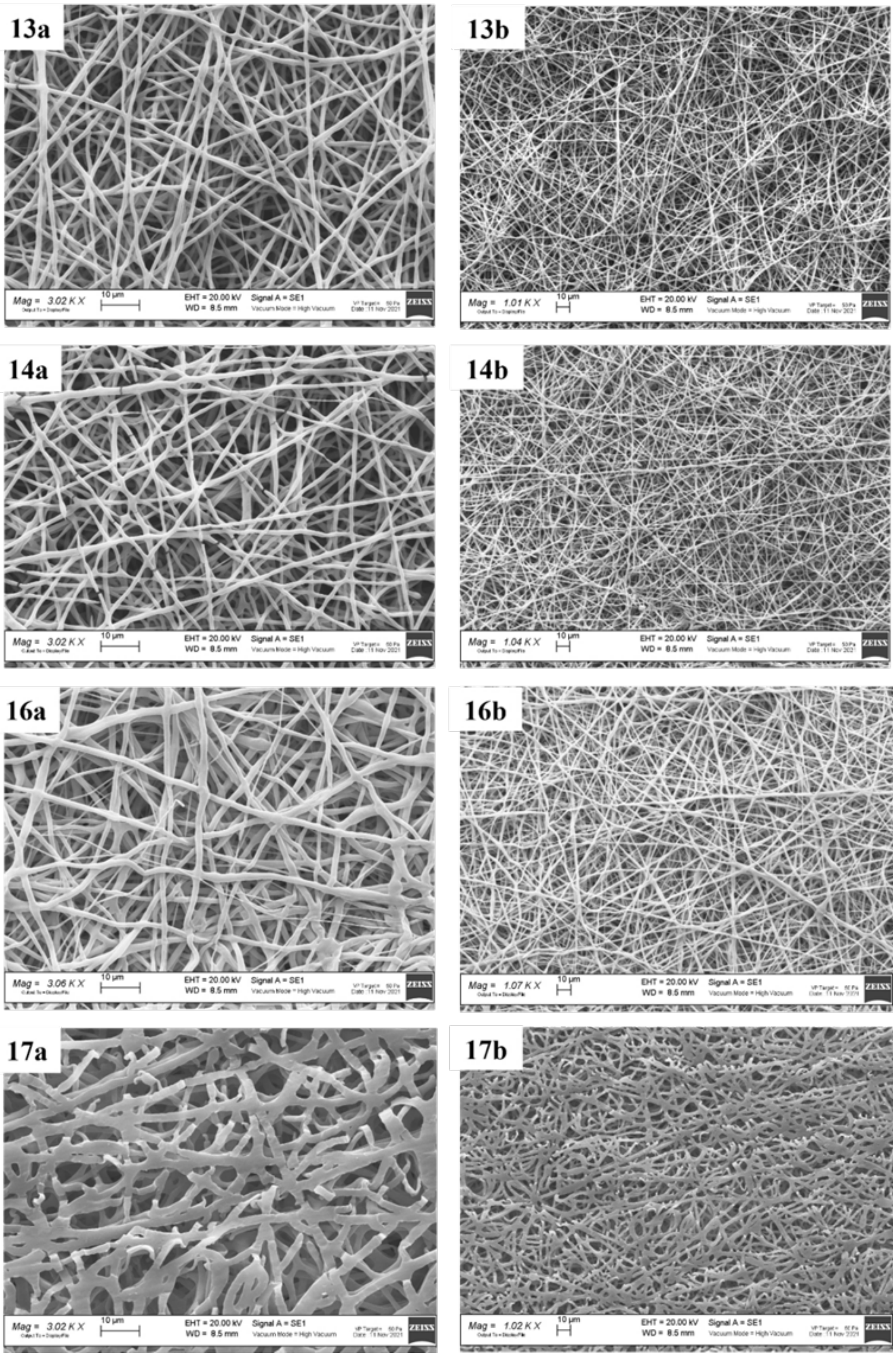

Figure S2. Scanning Electron Microscopy imaging at 3000x (a, left panel) and 1000x (b, right panel) magnification of electrospinning trials n. 13, 14, 16, 17 performed with PCL 14% w/v.

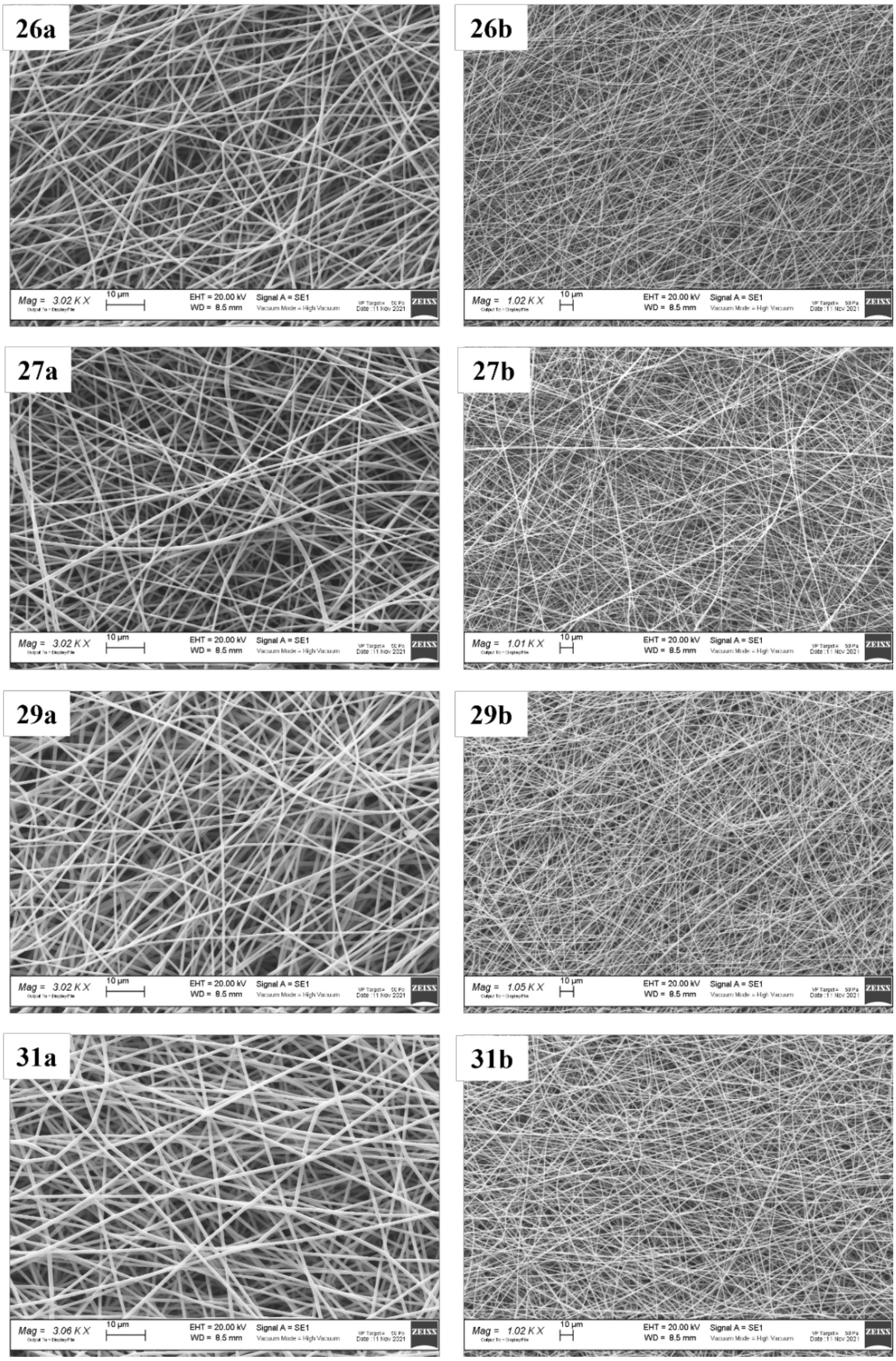

Figure S3. Scanning Electron Microscopy imaging at 3000x (a, left panel) and 1000x (b, right panel) magnification of electrospinning trials n. 26, 27, 29, 31 performed with PLGA 10% w/v.

S1.2.3. Solvent casting conditions optimization and scaffold morphological evaluation

Solvent casting/particulate leaching is a well-known technique for the easy preparation of highly porous polymeric scaffolds. It consists in the preparation of polymer solution and mineral or organic particles suspension, that is poured in a mould and allowed to dry through evaporation of the solvent. A polymer film entrapping the porogen is formed and the particle are removed by submerging the film in water, leaving only

the insoluble porous polymeric scaffold. This technique is suitable for the production of thin scaffolds, because thick polymer layers impede the complete removal of porogen particles (Ahmed et al., 2019). Several parameters influence the solvent casting/particulate leaching technique, including solution parameters, porogen characteristics and environmental parameters. Concerning solution parameters, the solvent plays a critical role in ensuring both dissolution of the polymer and drying of the film and should be therefore easily removed by evaporation from the scaffold; moreover, the solvent system and the polymer concentration influence the polymer solution rheological properties of , having an impact on the solution spreading in the mould and on the suspension stability preventing precipitation of the porogen particles. The porogen morphology and its particle size influence pore shape and dimensions , whereas its concentration determines the overall porosity and interconnectivity of the pores in the scaffold. Finally, the environmental temperature needs to be controlled, since it could affect the polymer solubility, the rheological properties of polymer solution, and the evaporation rate of the solvent (Eltom et al., 2019; Karki et al., 2016). Porogen type and amount of moulded polymer solution were optimized performing several solvent casting/particulate leaching trials, using 10% w/v PCL solutions, (**Table S2**) and evaluating the pores morphology and size, and porosity distribution by SEM imaging (**Figure S4**). Overall better results were obtained by casting 300 µL of solution (trials A, E and G) in the 2.5 mm diameter moulds, which formed a homogeneous polymer layer, whereas doubling the polymer solution to 600 µL (trials B and D) led to the formation of extremely large pores, probably due to the separation of polymer-particle suspension and enhanced an inhomogeneous shrinking of the polymer layer during drying. Moreover, when Glc and Lac were used as porogen (trials F and H), complete removal of the particle was impeded because of increased polymer film thickness. An increase of casting temperature to 40 °C (trials C and D) with the purpose to reduce viscosity and allow better spreading of the polymer solution, did not show any improvement of solvent casted scaffold morphology and was therefore discarded. **Figure S4** shows as trial E (Glc; 300 µL) and G (Lac; 300 µL) displayed suitable high microporosity at a 3000x magnification, but especially trial G showed at 600x magnification having also interconnected macropores thank to the bigger porogen particles. Trial A (NaCl; 300 µL) displayed a surface almost completely devoid of pores, except for very big pores that extend from the upper scaffold surface to the bottom and being therefore unsuitable as cell culturing and interaction support. Hence, due to the exceptional good macro- and microporosity of trial G (Lac; 300 µL) and the homogeneous distribution of porosity, spray dried lactose at a concentration of 0.35 g/mL of polymer solution, 61 µL/cm^2 of casted volume and a temperature of 20°C were selected as solvent casting parameters.

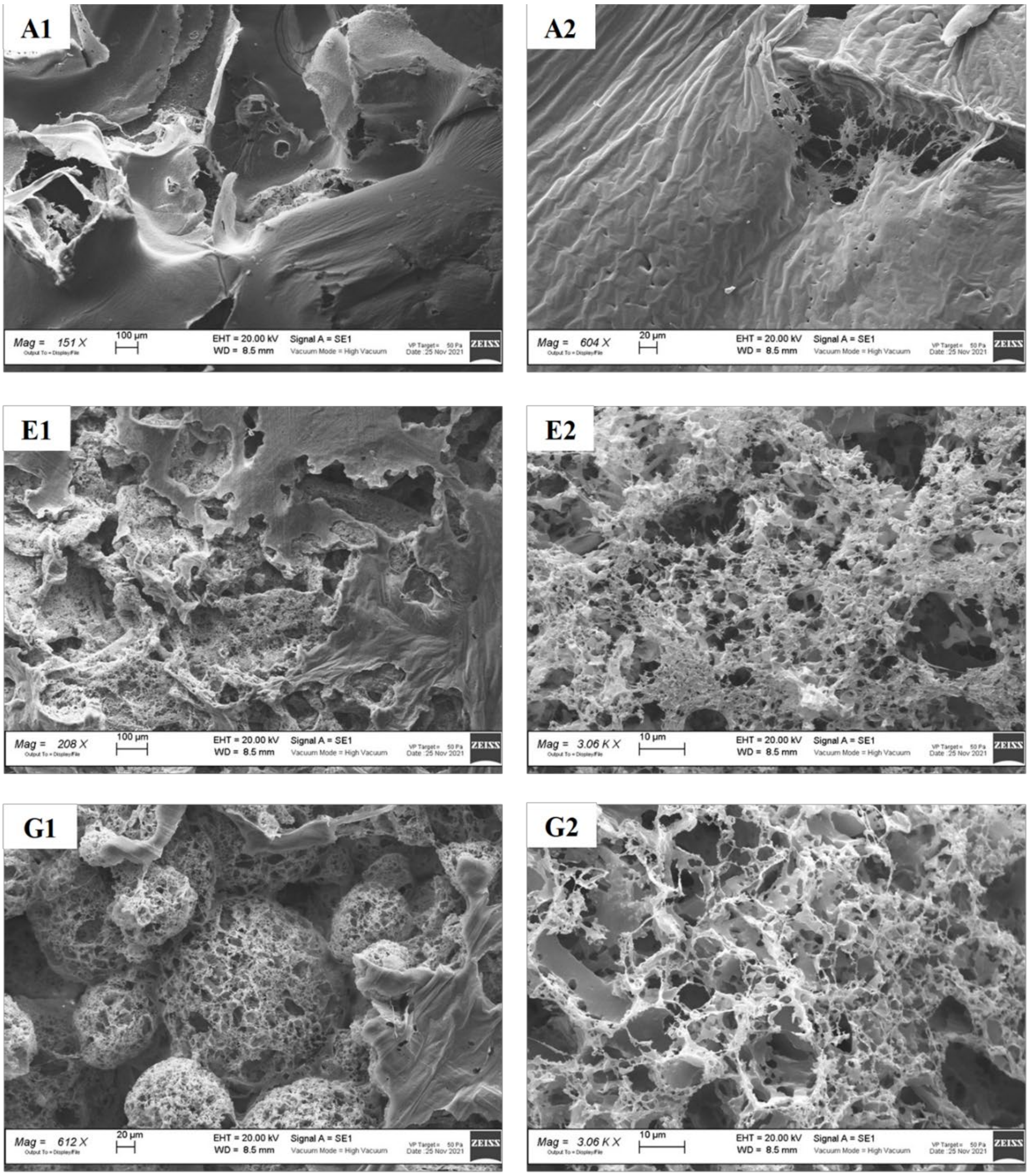

Figure S4: Scanning Electron Microscopy images of solvent casted scaffolds porogen trials A (porogen NaCl), E (porogen glucose) and G (porogen lactose) at different magnifications (see scaling bars).

S2. PLGA/PCL blend electrospun fibres diameter distribution

Fibre diameter frequency distribution of electrospun scaffolds ES1, ES2, ES3, ES4, ES5, and ES6 obtained from PCL/PLGA blends are shown in **Figure S5** and confirm that the increase of PLGA content in PCL predominant electrospun matrixes (ES2 and ES3) led to the formation of a heterogeneous population of fibres. On the contrary, the addition of PCL to PLGA predominant electrospun matrixes (ES5 and ES6) did not show an impact on the homogeneity of the fibres but had an influence on fibre diameter.

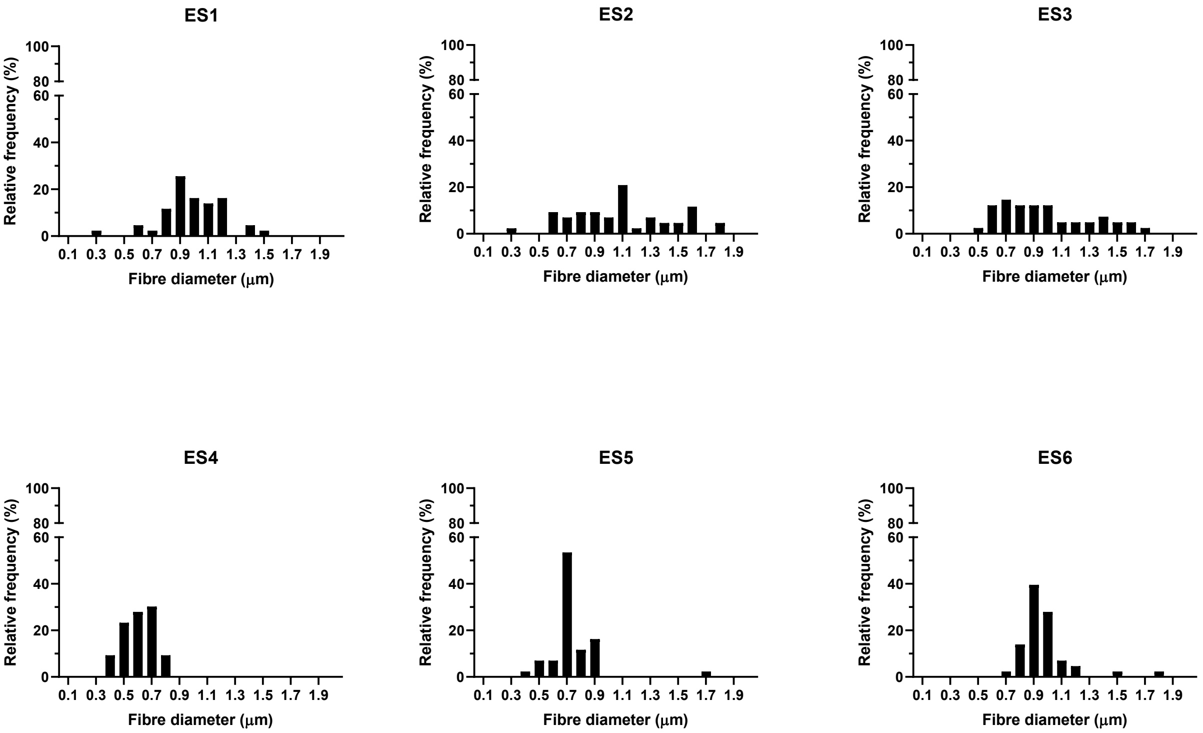

Figure S5: Frequency distribution of fibre diameter expressed in percentage of different PCL/PLGA blend electrospun samples: ES1 (PCL100%), ES2 (PCL95%+PLGA5%), ES3 (PCL85%+PLGA15%), ES4 (PLGA100%), ES5 (PLGA95%+PCL5%), ES6 (PLGA85%+PCL15%).

Supplementary Material Bibliography

Ahmed, S., Chauhan, V.M., Ghaemmaghami, A.M., Aylott, J.W., 2019. New generation of bioreactors that advance extracellular matrix modelling and tissue engineering. Biotechnol. Lett. 41, 1–25. https://doi.org/10.1007/s10529-018-2611-7

Chiesa, E., Tottoli, E.M., Giglio, A., Conti, B., Rosalia, M., Rizzi, L.G., Dorati, R., Genta, I., 2022. Graphene Nanoplatelets-Based Textured Polymeric Fibrous Fabrics for the Next-Generation Devices. Polymers (Basel). https://doi.org/10.3390/polym14245415

Eltom, A., Zhong, G., Muhammad, A., 2019. Scaffold Techniques and Designs in Tissue Engineering Functions and Purposes: A Review. Adv. Mater. Sci. Eng. 2019, 3429527. https://doi.org/10.1155/2019/3429527

Haider, A., Haider, S., Kang, I.-K., 2018. A comprehensive review summarizing the effect of electrospinning parameters and potential applications of nanofibers in biomedical and biotechnology. Arab. J. Chem. 11, 1165–1188. https://doi.org/https://doi.org/10.1016/j.arabjc.2015.11.015

Ibrahim, H.M., Klingner, A., 2020. A review on electrospun polymeric nanofibers: Production parameters and potential applications. Polym. Test. 90, 106647. https://doi.org/https://doi.org/10.1016/j.polymertesting.2020.106647

Karki, S., Kim, H., Na, S.-J., Shin, D., Jo, K., Lee, J., 2016. Thin films as an emerging platform for drug

delivery. Asian J. Pharm. Sci. 11, 559–574. https://doi.org/https://doi.org/10.1016/j.ajps.2016.05.004

Particle size distribution estimation by analytical sieving, 2022. , in: European Pharmacopoeia. Council of Europe's European Directorate for the Quality of Medicines and Health Care, Strasbourg, pp. 3649–51.

Shaw, M.T., 2016. On estimating the zero-shear-rate viscosity: Tests with PIB and PDMS, in: AIP Conference Proceedings. AIP Publishing LLC, p. 70011.
